# Vitamin D_4_ in Mushrooms

**DOI:** 10.1371/journal.pone.0040702

**Published:** 2012-08-03

**Authors:** Katherine M. Phillips, Ronald L. Horst, Nicholas J. Koszewski, Ryan R. Simon

**Affiliations:** 1 Biochemistry Department, Virginia Tech, Blacksburg, Virginia, United States of America; 2 Heartland Assays, Inc., Ames, Iowa, United States of America; 3 Department of Biomedical Sciences, College of Veterinary Medicine, Iowa State University, Ames, Iowa, United States of America; 4 Cantox Health Sciences International, Mississauga, Ontario, Canada; Clermont Université, France

## Abstract

An unknown vitamin D compound was observed in the HPLC-UV chromatogram of edible mushrooms in the course of analyzing vitamin D_2_ as part of a food composition study and confirmed by liquid chromatography-mass spectrometry to be vitamin D_4_ (22-dihydroergocalciferol). Vitamin D_4_ was quantified by HPLC with UV detection, with vitamin [^3^H] itamin D_3_ as an internal standard. White button, crimini, portabella, enoki, shiitake, maitake, oyster, morel, chanterelle, and UV-treated portabella mushrooms were analyzed, as four composites each of a total of 71 samples from U.S. retail suppliers and producers. Vitamin D_4_ was present (>0.1 µg/100 g) in a total of 18 composites and in at least one composite of each mushroom type except white button. The level was highest in samples with known UV exposure: vitamin D enhanced portabella, and maitake mushrooms from one supplier (0.2–7.0 and 22.5–35.4 µg/100 g, respectively). Other mushrooms had detectable vitamin D_4_ in some but not all samples. In one composite of oyster mushrooms the vitamin D_4_ content was more than twice that of D_2_ (6.29 vs. 2.59 µg/100 g). Vitamin D_4_ exceeded 2 µg/100 g in the morel and chanterelle mushroom samples that contained D_4_, but was undetectable in two morel samples. The vitamin D_4_ precursor 22,23-dihydroergosterol was found in all composites (4.49–16.5 mg/100 g). Vitamin D_4_ should be expected to occur in mushrooms exposed to UV light, such as commercially produced vitamin D enhanced products, wild grown mushrooms or other mushrooms receiving incidental exposure. Because vitamin D_4_ coeluted with D_3_ in the routine HPLC analysis of vitamin D_2_ and an alternate mobile phase was necessary for resolution, researchers analyzing vitamin D_2_ in mushrooms and using D_3_ as an internal standard should verify that the system will resolve vitamins D_3_ and D_4_.

## Introduction

Vitamin D is a 9,10-secosteroid and 6 forms have been identified [1]_._ Vitamin D_2_ (9,10-seco(5*Z*,7*E*)-5,7,10(19),22-ergostatetraene-3β-ol; ergocalciferol) and vitamin D_3_ (9,10-seco(5*Z*,7*E*)-5,7,10(19)cholestatriene-3β-ol; cholecalciferol) are the predominant forms of vitamin D relevant to human nutrition. Vitamin D_3_ originates from animal sources, and vitamin D_2_ is derived predominantly from fungi, such as yeast [2], [3]. The importance of vitamin D in bone (calcium homeostasis) is well established, and vitamin D has been the subject of increased attention in recent years for its role in muscle function, immunology, heart and cardiovascular disease, cancer, and insulin secretion [4], [5], [6], [7], [8]. A primary source of vitamin D_3_ in humans and many animals occurs from the conversion of 7-dehydrocholesterol in the epidermis to vitamin D_3_ during exposure to ultraviolet (UV) radiation present in sunlight [2]. Oily fish and fish liver oils are naturally rich dietary sources of vitamin D_3_. Other foods in the U.S. marketplace are fortified (typically with vitamin D_3_), including milk, cheeses, yogurts, cereals, margarines, and orange juice.

Mushrooms are a natural source of vitamin D_2._ The vitamin D_2_ content of mushrooms can be increased dramatically by UV irradiation, whereby ergocalciferol is formed from ergosterol [9], [10], [11], [12], [13]. Recent analyses conducted on ten types of mushrooms sampled from the U.S. marketplace showed vitamin D_2_ concentrations between 0.03–63.2 μg/100 g (1.2–2528 IU/100 g) fresh weight, with the highest levels in mushrooms exposed to UV during production [14]. Ergosterol is also found in yeast and other fungi [15], and vitamin D_2_ is produced industrially by UV irradiation of yeast [3]. Vitamin D_2_ is included in some dietary supplements and fortified foods, particularly vegetarian products.

The occurrence of vitamers other than D_3_ and D_2_ in the food supply has not been widely reported in the literature, nor have their nutritional value and biological effects. In the available studies evaluating the vitamin D content in different mushroom species (including Mattila et al. [16], [17], [18], Rangel-Castro et al. [19], Teichmann et al. [13]), no vitamers other than D_2_ have been reported. In our recent analysis of the vitamin D_2_ and sterol content of ten types of mushrooms [14] a second peak having a UV spectrum consistent with vitamin D was present in the HPLC chromatogram of many samples and occurred at a relatively high level in mushrooms that had been exposed to UV light. The vitamin D_4_ precursor ergosta-5,7-dienol (22,23-dihydroergosterol) was present in all samples. The purpose of this communication is to report on findings that support the identification of vitamin D_4_ in mushrooms, and the vitamin D_4_ content of ten types of mushrooms.

**Figure 1:**
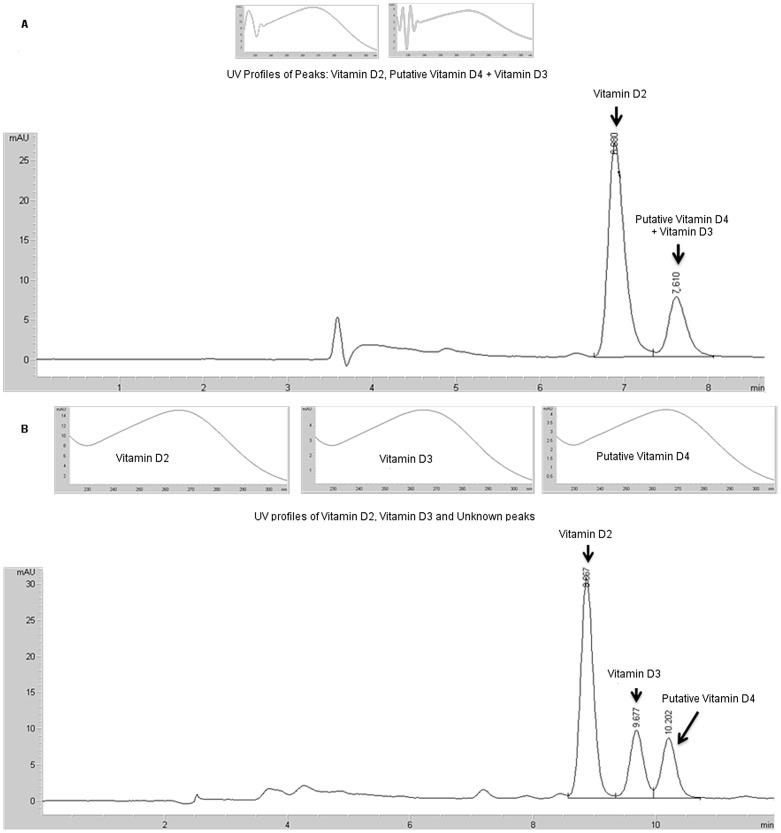
HPLC chromatograms and UV spectra of vitamin D components in a mixed mushroom extract. Chromatography on a Vydac® ODS column developed using (**A**) acetonitrile:methylene chloride (70∶30) (the solvent system used previously for quantitation of vitamin D_2_ [14]), showing co-migration of the putative vitamin D_4_ with vitamin D_3_ in this system; (**B**) developed with acetonitrile:methanol (1∶1) mobile phase, showing separation of the peak containing putative vitamin D_4_ and vitamin D_3_ into two components.

**Figure 2:**
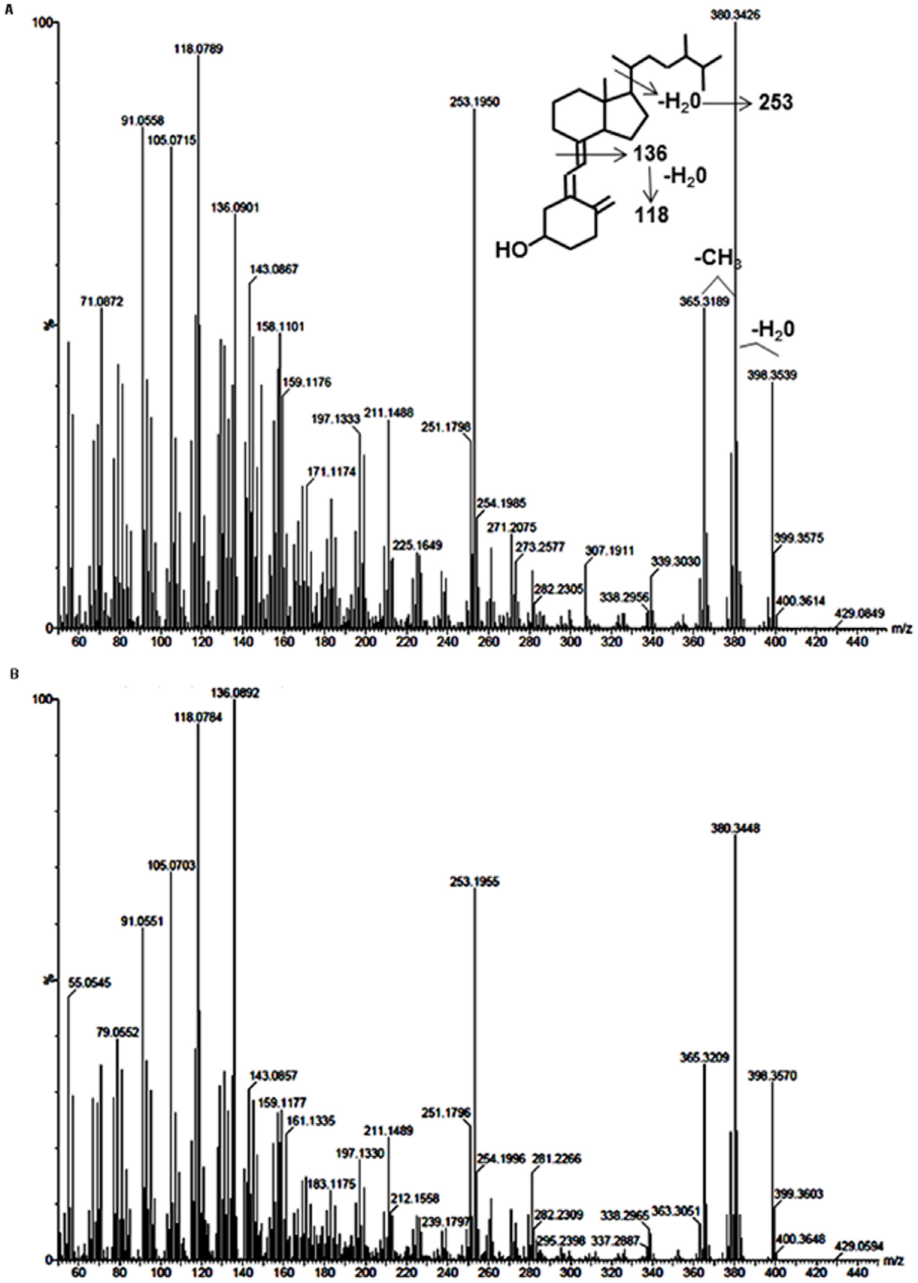
High resolution mass spectral comparison of putative vitamin D_4_ isolated from mushroom. (**A**) Spectrum of HPLC-purified mushroom isolate corresponding to vitamin D_4_ with structure and breakdown products highlighted. (**B**) Spectrum of vitamin D_4_ standard.

## Materials and Methods

### General experimental procedures

Reagents and standards for extraction and analysis of vitamin D and sterols were as described previously [14]. Authentic vitamin D_4_ (manufacturer's specified purity, 98.9% by TLC9) was procured from Lanospharma Laboratories Co., Ltd. (Chongqing, China). Ergosterol and *N*,*O*-bis(trimethylsilyl)trifluoroacetamide (BSTFA) were purchased from Sigma-Aldrich Corp. (St. Louis, MO).

### Samples

Samples of white button, crimini, portabella, enoki, shiitake, maitake, oyster, morel, UV-treated portabella, and chanterelle mushrooms were the same as described in detail in the previous report on vitamin D_2_ and sterols [14] and comprised a total 71 original samples analyzed as four composites of each type of mushroom (two for chanterelle).

**Figure 3:**
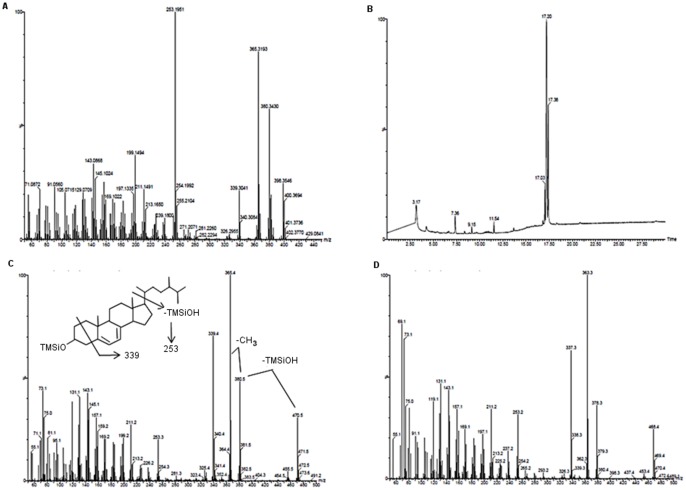
Spectral analysis of putative dihydroergosterol in a mushroom isolate. (**A**) High resolution mass spectrum of purified mushroom isolate corresponding to dihydroergosterol. (**B**) Gas chromatogram of products obtained following derivatization of the purified mushroom isolate with N,O-bis(trimethylsilyl)trifluoroacetamide (BSTFA). (**C**) Low resolution GC-MS of derivatized mushroom product at t = 17.20 min corresponding to dihydroergosterol with structure and breakdown products highlighted. (**D**) Low resolution GC-MS of commercially available ergosterol standard following derivatization with BSTFA.

**Table 1:** Vitamin D_4_ and pre-vitamin D_4_ (22,23-dihydroergosterol; ergosta-5,7-dienol) content of ten types of mushrooms.

					Vitamin D_4_				22,23-Dihydroergosterol	
Mushroom	Scientific name	NDB no.^a^	Com- posite^b^	Moisture (g/100g)	µg/100g fresh weight^c^	Mean	SD	Std Err	mg/100g fresh weight	Mean
White button	*Agaricus bisporus*	11260	1	92.85	–	–^B^	–	–	5.97	6.03 ^B,C^
			2	92.81	–				5.79	
			3	92.35	–				5.86	
			4	92.47	–				6.49	
Enoki	*Flammulina veluptipes*	11950	A1	87.68	–	0.10 ^B^	0.21	0.10	17.0	16.5 ^A^
			A2	88.47	–				18.0	
			G1	88.28	0.41				17.0	
			1	89.30	–				13.8	
Shiitake	*Lentinus edodes*	11238	1	86.90	0.27	0.51 ^B^	0.48	0.24	7.31	6.51 ^B,C^
			2	91.41	0.67				7.25	
			3	90.53	1.11				6.15	
			A1	90.11	–				5.34	
Maitake	*Grifola frondosa*	11993	A1	88.37	–	14.5 ^A^	17.5	8.76	8.90	6.34 ^B,C^
			A2	88.59	–				9.00	
			C1	92.30	35.4				3.53	
			C2	91.92	22.5				3.92	
Oyster	*Pleurotus ostreatus*	11987	A1	89.70	0.81	1.77 ^AB^	3.00	1.52	8.55	8.89 ^B^
			1	88.77	–				11.7	
			2	90.38	6.29				8.16	
			3	90.54	–				7.13	
Crimini	*Agaricus bisporus*	11266	1	91.92	–	0.31 ^B^	0.61	0.31	5.25	5.92 ^B,C^
			2	91.22	1.22				6.11	
			A1	93.08	–				5.42	
			B1	92.07	–				6.92	
Portabella	*Agaricus bisporus*	11265	1	90.96	–	0.14^B^	0.27	0.14	6.75	6.18 ^B,C^
			2	92.22	–				5.45	
			3	91.29	0.55				6.53	
			4	91.25	–				5.97	
Portabella, uv treated	*Agaricus bisporus*	11998	A1	94.86	0.20	3.62 ^AB^	3.22	1.61	4.57	4.70 ^C^
			A2	95.12	1.66				3.94	
			B1	94.76	7.05				5.10	
			B2	93.68	5.56				5.20	
Chanterelle	*Cantharellus californicus or C. cibarius*	11239	D1	91.09	0.82	1.62 ^AB^	1.13	0.80	5.23	4.49 ^C^
			D2	88.61	2.42				3.75	
Morel	*Morchella spp.*	11240	E1	89.46	2.36	1.13 ^B^	1.31	0.65	7.13	5.79 ^B,C^
			E2	90.38	2.15				5.75	
			F1	89.44	–				5.31	
			F2	89.18	–				4.98	

a Database entry number from United States Department of Agriculture (USDA) National Nutrient Database for Standard Reference [53]; ^b^ Composites are combinations of samples from statisitical sampling locations in the U.S., or retail suppliers, as described in Phillips et al. [14]. Composites designated with the same capital letter were from the same supplier. ^c^ – indicates less than the limit of detection (0.1 μg/100 g fresh weight).

### Extraction and analysis of sterols and vitamin D

Sterols and vitamin D_2_ were quantified as described previously [14]. Sterols were determined as the trimethylsilyl ether (TMS) derivatives, by gas chromatography with flame ionization detection after alkaline saponification of total lipid extracts, with gas chromatography-mass spectrometry (GC-MS) to confirm component identities. Vitamin D_4_ was quantified using high-performance liquid chromatography (HPLC) with UV detection and [^3^H]vitamin D_3_ as the internal standard as described previously for vitamin D_2_ [14], except using the HPLC conditions described below.

### Identification of the unknown

Mass spectrometry was performed at the High Resolution Mass Spectrometry Facility at the University of Iowa (Iowa City, IA) using a Waters GCT Premier (Waters Corp. Milford, MA). For solid probe high resolution mass spectrometry the ramp temperature used was 100°C/min. For GC-MS the column was a 30m DB-5ms and the ramp started at 170°C, then increased by 10°C per minute with a final temperature of 300°C that was held for 15 minutes. BSTFA derivatization for GC-MS was performed by re-suspending dry samples or standards in a 1∶1 mixture of BSTFA:methylene chloride, warming at 40°C for 60 minutes followed by direct injection of an aliquot of a given mixture onto the GC-MS column.

**Figure 4:**
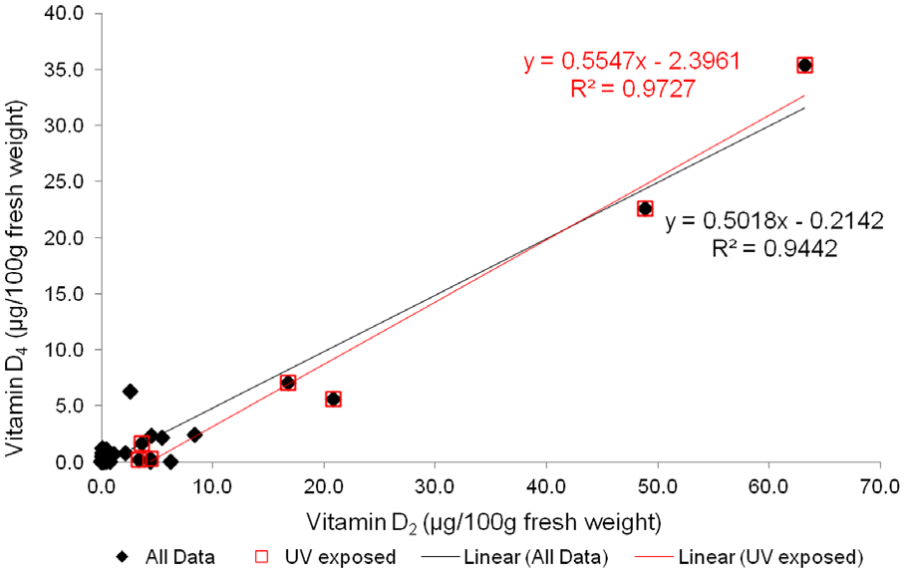
Relationship between the vitamin D_4_ and vitamin D_2_ concentrations in ten types of mushrooms (**Table 1**). Data for vitamin D_2_ were previously reported [14].

**Table 2:** Comparison of assayed concentrations of ergosterol (vitamin D_2_ precursor) and 22,23-dihydroergosterol (vitamin D_4_ precursor) in white and brown button mushrooms.

		Range (μg/100g dry weight)	
Component		This study	Shao et al. [31] a
Ergosterol	White button	740–795	563–681
	Brown buttonc	725–821	475–938
22,23-Dihydroergosterol	White button	77–86	71–95 b
	Brown buttonc	65–87	42–65 b
22,23-Dihydroergosterol	White button	10.0–11.2	11.2–14.0
(as percent of ergosterol)	Brown buttonc	7.9–12.0	6.9–10.5

In this study for four samples of each type, and as reported by Shao et al. [31] for one sample at each of three stages of maturity for each mushroom type.

a values show the sum of the concentrations in the separately assayed stems and caps.

b reported as “ergosterol analogue”.

c crimini.

### Quality control

A sample of a mushroom control composite previously described [14], that comprised approximately 50% portabella mushrooms and 50% vitamin D enhanced (UV-treated) portabella mushrooms, was analyzed with each batch of samples and used to monitor run-to-run precision. Validation of recovery of vitamin D_2_ as described in a previous communication [14] was assumed to apply to the extraction of vitamin D_4_. The GC-MS analyses described above verified the identity of the analyte peaks.

### Data analysis

Means and standard deviations were calculated using Microsoft® Office Excel (Professional Plus edition, 2010; Microsoft Corporation, Redmond, WA), and analysis of variance (α = 0.05) and pairwise comparison of means using the Student-Newman-Keuls test with a 95% confidence interval were performed with XLSTAT (version 2011.2.06; Addinsoft, New York, NY).

**Figure 5:**
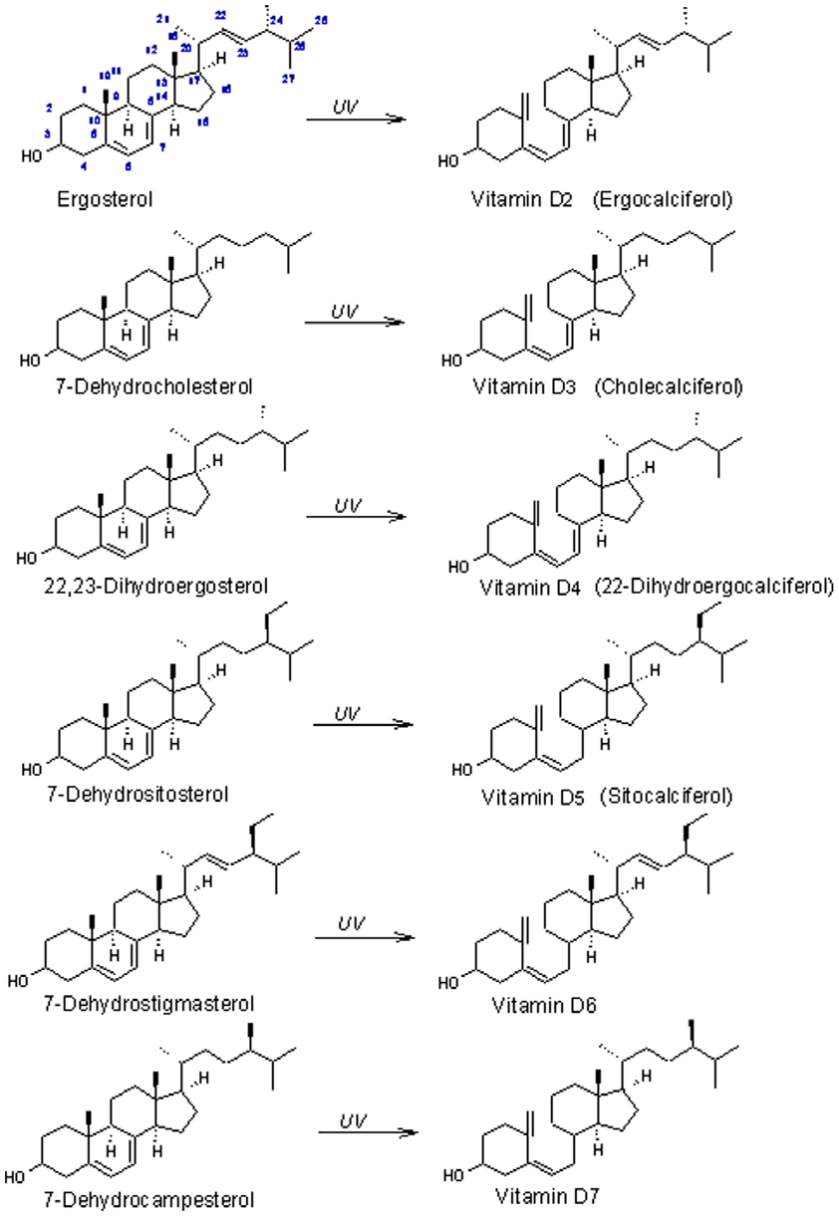
Structure of six forms of vitamin D their sterol precursors.

## Results

### Identification of vitamin D unknown in mushrooms

Initially the unknown vitamin D form observed in a variety of mushrooms in previous work [14] was thought to be vitamin D_3,_ because it eluted at the same retention time as a vitamin D_3_ standard chromatographed under the conditions that were being used for analysis of vitamin D_2_ and displayed the characteristic UV chromophore. Some literature reports were found on the presence of vitamin D_3_ in alfalfa, tomato, eggplant and zucchini leaves and some other plants have been reported [20], [21], [22], [23], but none on nutritional quantities of vitamin D_3_ or other forms besides D_2_ in mushrooms.

Figure 1 shows the high-performance liquid chromatography (HPLC) chromatogram of a mushroom extract containing the putative vitamin D_4_ and spiked with vitamin D_3._ Figure 1A shows the chromatogram from the solvent system routinely used for vitamin D analysis (acetonitrile/methylene chloride (70/30) as described by Phillips et al. [14]; Figure 1B shows the separation of the vitamin D_3_ and putative vitamin D_4_ into two components using an alternate solvent system (acetonitrile:methanol, 1∶1), confirming the component was not D_3_. The unknown was hypothesized to be vitamin D_4_ (22-dihydroergocalciferol) because it co-eluted with an authentic vitamin D_4_ using the alternative solvent system and because its precursor is present in mushrooms. Although there have been no previous literature reports of vitamin D_4_ in mushrooms, vitamin D_4_ (22,23-dihydroergocalciferol;9,10-seco(5*Z*,7*E*)-5,7,10(19)-ergostatriene-3β-ol) is the product of UV irradiation of 22,23-dihydroergosterol, analogous to the formation of vitamin D_2_ from ergosterol. 22,23-dihydroergosterol (ergosta-5,7-dienol) was present in ten types of mushrooms, as previously reported [14]. Therefore it seemed reasonable to presume conversion of some portion of the 22,23-dihydroergosterol to vitamin D_4,_ and mass spectral studies were conducted to confirm the identity.

### Mass spectral confirmation

Material was collected from the putative vitamin D_4_ peak of a mixture of mushroom types and analyzed by high resolution mass spectrometry and compared with an authentic vitamin D_4_ standard run under identical conditions. As seen in Figure 2A, the mushroom compound produced a parent molecular ion at m/z 398.3539, in good agreement with the calculated mass value of 398.3549 for vitamin D_4_. Losses of water and a methyl group are readily apparent (m/z 380.3426 and 365.3189). The prominent peak at 253.1950 corresponds to loss of the vitamin D_4_ side chain in combination with a water molecule, while peaks at 136.0901 and 118.0789 are characteristic for cleavage of the secosteroid structure and subsequent water loss. All of these fragments were also observed with the authentic vitamin D_4_ compound when subjected to the same high resolution analysis (Figure 2B). In addition, low resolution mass spectrometry of TMS-derivatized samples of both the mushroom isolate and vitamin D_4_ standard produced analogous mass spectral fragmentations, with parent ions of m/z 470.5 (data not shown), thus verifying the presence of a single hydroxyl moiety and further corroborating the identity of this compound from the mushroom isolate as vitamin D_4_.

In a similar manner, high resolution mass spectrometry was also performed on the purported 22,23-dihydroergosterol collected from the mixed mushroom sample; however, in contrast the spectra revealed the presence of at least 2 compounds, with molecular ions evident at m/z 398.3546 and 400.3694 (Figure 3A). The lower mass was in agreement with the prediction for 22,23-dihydroergosterol (C_28_H_46_O; calculated value 398.3549), while the higher mass suggested an additional saturation of a diene bond, presumably of a 22,23-dihydroergosterol-like molecule (C_28_H_48_O; calculated value of 400.3705). Because of the apparent complexity of the sample, the mixture was derivatized with BSTFA and subjected to gas chromatography-mass spectrometry (GC-MS). As seen in Figure 3B, the gas chromatogram of the TMS-derivatized mushroom isolate revealed the presence of 3 peaks. The major peak (17.2 min) produced a parent ion of m/z 470.5, in keeping with the derivatization of a single hydroxyl moiety and consistent with the expected ion mass for the TMS derivative of 22,23-dihydroergosterol (Figure 3C). Ions corresponding to loss of trimethylsilanol (m/z 380.5) followed by a methyl group (m/z 365.4) were readily apparent. The decrease of 131 mass units to produce the ion at m/z 339 is proposed to arise from fragmentation of the A-ring, most likely involving loss of C-2, C-3, C-4 and their substituents [24], [25]. Importantly, the presence of the m/z 253.3 ion, representing the core ring structure resulting from loss of the side chain and trimethylsilanol fragments indicates the additional saturation with hydrogen molecules occurred in the side chain. By way of comparison, an authentic ergosterol standard was similarly derivatized with BSTFA and subjected to GC-MS, which produced a single peak at 17.4 minutes (data not shown). As seen in Figure 3D the fragmentation pattern for derivatized ergosterol standard essentially paralleled that of the mushroom isolate, including the presence of the m/z 253.2 ion; except for the observed decrease in the molecular ion due to unsaturation of the side chain in the standard material. Thus, the data are consistent with the isolation of 22,23-dihydroergosterol from the mushroom extract. Finally, the other 2 peaks observed in the GC trace from the derivatized mushroom isolate (17.03 and 17.38 min) both produced parent ions at m/z 472 and fragments at m/z 255 (data not shown). As noted above, we suspect these may be isomers corresponding to additional saturation of one or the other of the diene bonds in the B-ring of 22,23-dihydroergosterol to produce, for instance, 22,23-dihydrobrassicasterol. Additional experiments will need to be performed to confirm these suspicions; however, the loss of the diene entity would explain the extent to which these compounds could co-migrate with the 22,23-dihydroergosterol and escape detection by HPLC utilizing an ultraviolet light detector to track the purification of the mushroom compounds.

The quantitative values for 22,23-dihydroergosterol that are reported were obtained in the previously reported GC and GC-MS analysis [14], which provided better resolution and eliminated the interference of the other components that were shown to coelute with 22,23-dihydroergosterol in the HPLC system.

### Vitamin D_4_ content of mushrooms

Table 1 summarizes the assayed concentration (fresh weight basis) of vitamin D_4_ and its precursor, 22,23-dihydroergosterol in ten types of mushrooms (white button, crimini, portabella, enoki, shiitake, maitake, oyster, morel, and UV-treated portabella, and chanterelle) sampled from retail outlets in the U.S. Overall, vitamin D_4_ was detected (>0.1 µg/100 g) in 18 of the total of 38 composites analyzed and was present at an average concentration of 5.2 µg/100 g. However there was wide variability between and within samples different types of mushrooms. There were 7 samples known to contain mushrooms that had been exposed to UV light during production: the Mushroom CC, the vitamin D enhanced portabella, and the two maitake composites from supplier G (Table 1). All of these samples contained vitamin D_4_, and in some the concentration was similar to or greater than that of vitamin D_2_ (previously reported in Phillips et al. [14]). The two maitake mushroom samples that were high in vitamin D_2_ (63.2 and 48.9 µg/100 g) were also high in vitamin D_4_ (35.4 and 22.5 µg/100 g, respectively). These mushrooms were presumed to have been exposed to UV light under the growing conditions reportedly used by this producer [26]. Of the mushrooms not known to have received UV exposure, vitamin D_4_ occurred in at least one composite of each type except white button. In oyster mushrooms the composite highest in vitamin D_2_ (2.59 µg/100 g) had a vitamin D_4_ content more than two-fold higher (6.29 µg/100 g). Vitamin D_4_ exceeded 2 µg/100 g in the morel and chanterelle mushroom samples that contained D_4_ (all but two morel composites).

Results for a total of 26 analyses of a control composite (Mushroom CC) across multiple assays provided an estimate of the analytical uncertainty in the vitamin D_4_ concentrations assayed in individual composites. The mean vitamin D_4_ concentration in the Mushroom CC was 0.14 µg/100g with a standard deviation of 0.042 µg/100 g (standard error, 0.008 µg/100 g). Greater precision at higher concentrations would be expected [27].

The presence of vitamin D_4_ in all mushrooms with known UV exposure but with no consistency in other samples suggests that vitamin D_4_ in mushrooms results from incidental or intentional UV exposure. Interestingly, Wang et al. [28] reported variability in the vitamin D level in lichens (*Cladina* spp.) as related to UV exposure at different latitudes. Figure 4 illustrates vitamin D_4_ concentration as a function of vitamin D_2_ concentration (previously reported [14]) in the 38 composites of ten types of mushrooms that were analyzed. Overall there was a positive correlation between vitamins D_4_ and D_2_. In a separate study of white button mushrooms subjected to controlled UV exposure [29], all of the UV-treated samples contained vitamin D_4_, with an average of 2.43 μg/100 g fresh weight (range 1.95–2.74), whereas the concentration was <0.1 μg/100 g in the unexposed mushrooms.

### Vitamin D_4_ precursor in mushrooms

The vitamin D_4_ precursor 22,23-dihydroergosterol was present in all mushroom composites (Table 1). The levels were not correlated with vitamin D_4_, but differed among species. Enoki mushrooms had a notably higher 22,23-dihydroergosterol content, with an average of 16.5 mg/100g compared to 4.49–8.89 mg/100 g in other types of mushrooms.

There have been other, limited reports on 22,23-dihydroergosterol in mushrooms, although the diversity in common nomenclature for sterols often makes the synonymous identity or close structural similarity among various sterols not readily apparent (see Moss [30] for detailed information on steroid nomenclature). 22-23-Dihydroergosterol [(24*R*)-24-methylcholesta-5,7-dien-3β-ol] is ergosta-5,7-dienol, and ergosta-5,7-dienol in wild and cultivated mushrooms [*Cantharellus cibarius* and *C. tubaeformis* (chanterelle), *Boletus edulis* (king bolete), *Lentinus edodes* (shiitake), *Pleurotus ostreatus* (oyster), and *Agaricus bisporus* (white button, brown button, crimini), portabella] was reported by Teichmann et al. [13]. Vitamin D_2_ levels were also analyzed in that study but no chromatograms from the vitamin D analysis were published, so it is not possible to determine if vitamin D_4_ may have been present. Shao et al. [31] recently reported the ergosterol content of stems and caps of white and brown button mushrooms at different stages of development and identified an “ergosterol analogue” in their HPLC analysis. This component is likely 22,23-dihydroergosterol based on comparison of the concentrations reported to those in the present study, and the fact that this component was identified in all samples of white and brown mushrooms in the present investigation. In the Shao et al. study [31] the sum of the concentration of the “ergosterol analogue” in the saponified extracts of the stems and caps was 0.71–0.95 mg/g dry wt and 0.42–0.65 mg/g dry wt in brown mushrooms (11.2–14.0% and 6.9–10.5% of the ergosterol concentration, respectively). These concentrations were similar to the averages of 0.82 mg/g dry wt and 0.75 mg/g dry wt for 22,23-dihydroergosterol (10.7% and 9.8% of the ergosterol concentration, respectively) in this study (Table 2).

## Discussion

The conjugated unsaturation at C-5 and C-7 in the B-ring is the key structural feature of sterols that are converted to vitamin D by UV irradiation. Figure 5 shows the sterol precursors of vitamin D compounds, which differ in the side chain at C-24 and the C22–23 bond. Excellent reviews are available on the metabolism and physiology of vitamin D [5], [32], [33]. Overall there is very little published on the physiological significance of vitamers other than D_3_ or their occurrence in foods and other natural products aside from vitamin D_2_ in mushrooms. Vitamin D_3_ and D_2_ are metabolized *in vivo* to the biologically active forms, 1α,25-dihydroxyvitamin D_3_ and D_2_ [22], [34]. The bioavailability of vitamin D_3_ is well established, and the bioavailability of vitamin D_2_ from mushrooms in humans has been shown to be comparable to that of a vitamin D_2_ supplement [35], [36]. Forms other than D_3_ have shown lower biological activity in vitamin D dependent cellular functions in some studies. DeLuca et al. [37] synthesized 22,23-[^3^H]vitamin D_4_ and compared its metabolism to 22,23-[^3^H]vitamin D_3_ in the rat. Vitamin D_4_ metabolites had a tissue distribution similar to vitamin D_3_ but were excreted more quickly but also appear to have lower toxicity in high doses compared to D_3_ [38].

The lower potential toxicity of vitamin D compounds other than D_3_ has spurred interest in their development as vitamin D analogs for use as potential pharmaceutical agents. The synthetic derivative of vitamin D_5_, 1α-hydroxyvitamin D_5_, has shown anti-tumor activity and been studied as an anti-cancer treatment [39], [40], [41]. Tachibana and Tsuji [42] found the metabolism of 1α,25-dihydroxyvitamin D_4_ to be similar to that of 1α,25-dihydroxyvitamin D_2_ in a study involving rats. Jones [43] has written an excellent review on vitamin D analogs, their pharmaceutical applications, and potential mechanisms of action.

Knowledge of the occurrence of lesser known forms of vitamin D and their sterol precursors, particularly in foods, herbal medicines, and materials that may be sources of these compounds is therefore valuable, given the potential value of vitamin D compounds. Some other organisms in which 22,23-dihydroergosterol (ergosta-5,7-dienol; 22-dihydroergosterol) has been reported include *Chlorella* species [44] and various yeasts and fungi [15], [45]. It has been found in *Mucor pusillus* [46], a source of a milk curdling protease used in cheese production. Interestingly, anobiid beetles have been shown to synthesize cholesterol from 22-dihydroergosterol supplied by symbiotic yeast, with 7-dehydrocholesterol (the precursor of vitamin D_3_) as the intermediate [47]. 22,23-dihydroergosterol and also 7-dehydrostigmasterol (another Δ5,7-sterol) and the precursor of vitamin D_6_ (Fig. 1) have been reported in *Trypanosoma cruzi*, the organism responsible for Chagas disease [48]. Vitamin D_5_ is the product of UV irradiation of 7-dehydrositosterol (Fig. 5). 7-dehydrositosterol has been reported in *Rauwolfia serpentina* (snakeroot), a plant commonly used in Chinese herbal medicine [49] and also in algae [50]. 7-dehydrocampesterol, the C-24 epimer of 22,23-dihydroergosterol [51] and the precursor to vitamin D_7_, has been found in *Crithidia fasciculate* [48] and in *Helianthus annuus* (sunflower) seed oil [52].

Because the vitamin D_4_ precursor 22,23-dihydroergosterol occurred in all mushrooms analyzed and vitamin D_4_ was found in approximately half of the samples overall and in all mushrooms with know UV exposure, its presence should be expected in mushrooms exposed to UV light in the commercial production of vitamin D enhanced products, or in wild grown or other mushrooms receiving incidental UV exposure.

Wide variability in the occurrence and vitamin D_4_ concentration in this relatively large sampling of mushrooms also suggests that the common practice of using vitamin D_3_ as an internal standard in the HPLC analysis of vitamin D_2_ in mushrooms will result in errors unless the separation of vitamins D_3_ and D_4_ by the chromatographic system is assured.

Further study of the biological activity of vitamin D_4_ is warranted, given its presence in many commonly consumed mushrooms.
