# Correction: Vitamin D_4_ in Mushrooms

**DOI:** 10.1371/journal.pone.0253992

**Published:** 2021-06-28

**Authors:** Katherine M. Phillips, Ronald L. Horst, Nicholas J. Koszewski, Ryan R. Simon

There are errors in sentences 3–7 of the Abstract. The correct text is as follows: Vitamin D_4_ was present (>0.01 µg/100 g) in a total of 18 composites and in at least one composite of each mushroom type except white button. The level was highest in samples with known UV exposure: vitamin D enhanced portabella, and maitake mushrooms from one supplier (0.02–0.7 and 2.25–3.54 µg/100 g, respectively). Other mushrooms had detectable vitamin D_4_ in some but not all samples. In one composite of oyster mushrooms the vitamin D_4_ content was about 25% of the vitamin D_2_ content (0.63 vs. 2.59 µg/100 g). Vitamin D_4_ exceeded 0.2 µg/100 g in the morel and chanterelle mushroom samples that contained D_4_, but was undetectable in two morel samples.

There are multiple errors in the section titled Vitamin D_4_ content of mushrooms. The corrected text is as follows.

Paragraph 1, Sentence 2: Overall, vitamin D_4_ was detected (>0.01 μg/100 g) in 18 of the total of 38 composites analyzed and was present at an average concentration of 0.52 µg/100 g.

Paragraph 1, Sentences 4–6: There were 7 samples known to contain mushrooms that had been exposed to UV light during production: the Mushroom CC, the vitamin D enhanced portabella, and the two maitake samples from supplier C (Table 1). All of these samples contained vitamin D_4_. The two maitake mushroom samples that were high in vitamin D_2_ (63.2 and 48.9 μg/100 g) were also high in vitamin D_4_ (3.54 and 2.25 μg/100 g, respectively). These mushrooms were found to have been exposed to UV light based on the growing conditions reported to be used by this producer [26].

Paragraph 1, Sentences 8–9: In oyster mushrooms the composite highest in vitamin D_2_ (2.59 μg/100 g) had a vitamin D_4_ content approximately 25% of D_2_ (0.63 μg/ 100 g). Vitamin D_4_ exceeded 0.2 μg/100 g in the morel and chanterelle mushroom samples that contained D_4_ (all but two morel composites).

Paragraph 2, Sentences 2–3: The mean vitamin D_4_ concentration in the Mushroom CC samples assayed in this study was 0.014 μg /100g with a standard deviation of 0.0042 μg /100 g (standard error, 0.0008 μg /100 g). Greater precision at higher concentrations would be expected [27].

There are errors in Table 1 and in Fig 4. In Table 1 and Fig 4, the values for vitamin D_4_ were off by a factor of 10. The Table 1 caption incorrectly reads “pre-vitamin D_4_” instead of “pro-vitamin D_4_”. Please see the corrected Table 1, Table 1 caption, and Fig 4 below.

**Fig 4:**
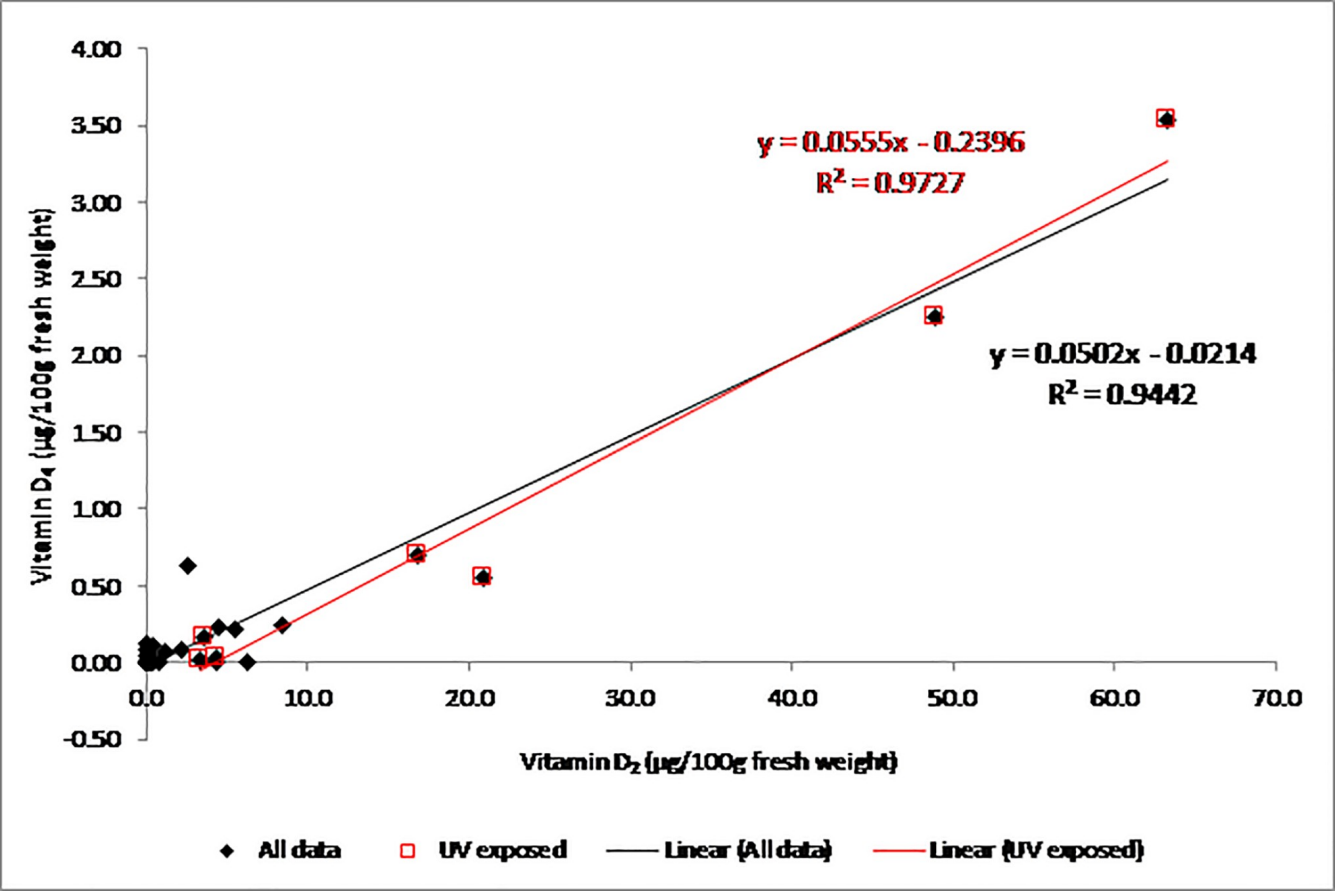
Relationship between the vitamin D4 and vitamin D2 concentrations in ten types of mushrooms (Table 1). Data for vitamin D2 were previously reported [14].

**Table 1:** Vitamin D_4_ and pro-vitamin D_4_ (22,23-dihydroergosterol; ergosta-5,7-dienol) content of ten types of mushrooms.

					Vitamin D_4_				22,23-Dihydroergosterol	
Mushroom	Scientific name	NDB no.^a^	Com-posite^b^	Moisture (g/100g)	µg/100g fresh weight^c^	Mean	SD	Std Err	mg/100g fresh weight^c^	Mean
White button	*Agaricus bisporus*	11260	1	92.85	-	-	-	-	5.97	6.03 ^B,C^
	* *		2	92.81	-				5.79	
			3	92.35	-				5.86	
	* *		4	92.47	-				6.49	
Enoki	*Flammulina veluptipes*	11950	A1	87.68	-	0.01 ^B^	0.02	0.01	17.0	16.5 ^A^
	* *		A2	88.47	-				18.0	
			G1	88.28	0.04				17.0	
	* *		1	89.30	-				13.8	
Shiitake	*Lentinus edodes*	11238	1	86.90	0.03	0.05 ^B^	0.05	0.02	7.31	6.51 ^B,C^
	* *		2	91.41	0.07				7.25	
			3	90.53	0.11				6.15	
	* *		A1	90.11	-				5.34	
Maitake	*Grifola frondosa*	11993	A1	88.37	-	1.45 ^A^	1.75	0.88	8.90	6.34 ^B,C^
	* *		A2	88.59	-				9.00	
			C1	92.30	3.54				3.53	
	* *		C2	91.92	2.25				3.92	
Oyster	*Pleurotus ostreatus*	11987	A1	89.70	0.08	0.18 ^A,B^	0.30	0.15	8.55	8.89 ^B^
	* *		1	88.77	-				11.7	
			2	90.38	0.63				8.16	
	* *		3	90.54	-				7.13	
Crimini	*Agaricus bisporus*	11266	1	91.92	-	0.03 ^B^	0.06	0.03	5.25	5.92 ^B,C^
	* *		2	91.22	0.12				6.11	
			A1	93.08	-				5.42	
	* *		B1	92.07	-				6.92	
Portabella	*Agaricus bisporus*	11265	1	90.96	-	0.01 ^B^	0.03	0.01	6.75	6.18 ^B,C^
	* *		2	92.22	-				5.45	
			3	91.29	0.05				6.53	
	* *		4	91.25	-				5.97	
Portabella, uv treated	*Agaricus bisporus*	11998	A1	94.86	0.02	0.36 ^A,B^	0.32	0.16	4.57	4.70 ^C^
	* *		A2	95.12	0.17				3.94	
			B1	94.76	0.70				5.10	
	* *		B2	93.68	0.56				5.20	
Chanterelle	*Cantharellus californicus or C*. *cibarius*	11239	D1	91.09	0.08	0.16 ^A,B^	0.11	0.08	5.23	4.49 ^C^
	* *		D2	88.61	0.24				3.75	
Morel	*Morchella spp*.	11240	E1	89.46	0.24	0.11 ^B^	0.13	0.07	7.13	5.79 ^B,C^
	* *		E2	90.38	0.22				5.75	
			F1	89.44	-				5.31	
	* *		F2	89.18	-				4.98	

^a^Database entry number from United States Department of Agriculture (USDA) National Nutrient Database for Standard Reference [53];

^b^ Composites are combinations of samples from statistical sampling locations in the U.S., or retail suppliers, as described in Phillips et al. [14]. Composites designated with the same capital letter were from the same supplier.

^c^—indicates less than the limit of detection (0.01 µg/100 g fresh weight).
